# Inheritance of Some Salt Tolerance-Related Traits in Bread Wheat (*Triticum aestivum* L.) at the Seedling Stage: A Study of Combining Ability

**DOI:** 10.3390/plants14060911

**Published:** 2025-03-14

**Authors:** Toka Hadji, Mouad Boulacel, Awatef Ghennai, Maroua Hadji, Fethi Farouk Kebaili, Chermen V. Khugaev, Olga D. Kucher, Aleksandra O. Utkina, Alena P. Konovalova, Nazih Y. Rebouh

**Affiliations:** 1 Laboratory of Development and Valorization of Plant Genetic Resources, Department of Plant Biology, University of Mentouri Brothers Constantine 1, Route de Ain El Bey, Constantine 25017, Algeria; mouad.boulassel@umc.edu.dz; 2 Department of Biological Engineering, Institute of Technology, Larbi Ben M’Hidi University, P.O. Box 358, Oum El Bouaghi 04000, Algeria; ghennai.awatif@univ-oeb.dz; 3 Biodiversity and Biotechnology Techniques of Plant Resources Valorization Laboratory, SNV Department, Faculty of Sciences, University of M’sila, M’sila 28000, Algeria; maroua.hadji@univ-msila.dz; 4 Laboratory of Microbiological Engineering and Application, Department of Biochemistry and Molecular and Cellular Biology, Faculty of Nature and Life Sciences, University of Mentouri Brothers Constantine 1, P.O. Box 325, Ain El Bey Way, Constantine 25017, Algeria; kff.fethi@gmail.com; 5 The General Directorate of National Security, The Sub-Directorate of Scientific and Technical Police, The Regional Laboratory of Scientific Police, Ain El Bey City, Constantine 25017, Algeria; 6 Department of Environmental Management, Institute of Environmental Engineering, RUDN University, 6 Miklukho-Maklaya St., 117198 Moscow, Russia; hugai-mvd@yandex.ru (C.V.K.); kucher-od@rudn.ru (O.D.K.); utkina-ao@rudn.ru (A.O.U.); psareva-ap@rudn.ru (A.P.K.)

**Keywords:** abiotic stress, Algerian oasis wheat, hybridization, general combining ability (GCA), specific combining ability (SCA)

## Abstract

The worldwide rise in soil salinization is among the most critical consequences of climate change, posing a significant threat to food security. Wheat (*Triticum aestivum* L.), a staple crop of paramount importance worldwide, encounters significant production limitations due to abiotic stressors, particularly salinity. Consequently, the development and cultivation of salt-tolerant wheat genotypes have emerged as an essential strategy to sustain agricultural productivity and safeguard global food security. The aim of the present study was to investigate the effect of salinity (150 mM) on the performance and combining ability of 10 hybrid combinations (F2) and their parents that were obtained through a line × tester mating design at the seedling stage. Morphological, physiological, and biochemical traits were assessed under both control and salt-stress conditions. Among the assessed traits, SFW emerged as the strongest predictor of salt tolerance, demonstrating the highest correlation with MFVS and the greatest contribution in the regression model. The results highlighted distinct responses among the studied genotypes. Hybrid H5 demonstrated particular promise, surpassing the performance of the superior parent for Na^+^, K^+^, K^+^/Na^+^ and proline (Pro). Furthermore, tester T1 emerged as a good combiner for proline (Pro), total soluble sugars content (Sug), chlorophyll content (Chl) and root length (RL) under saline conditions. In contrast, under control conditions, line L1 and testers T2, T3, and T5 exhibited superior performance, demonstrating significant general combining ability (GCA) effects for four traits simultaneously. Hybrid H4 emerged as outstanding under salt stress, exhibiting favorable specific combining ability (SCA) effects for Na^+^, K^+^/Na^+^ ratio, root length (RL), relative water content (RWC), and total soluble sugars content (Sug). Under normal conditions, hybrids H7 and H10 exhibited significantly superior performance across three traits simultaneously. Non-additive genetic effects predominantly influenced the studied traits under both conditions. The parental and hybrid combinations show promise for incorporation into breeding programs designed to improve salt tolerance under the specific conditions studied.

## 1. Introduction

Wheat dominates as the primary food source across North Africa and Central and West Asia by providing nearly half of the average person’s daily calories, with regional consumption being the highest wheat intake worldwide [1]. However, with a growing population, and if wheat continues to play a similar role in global diets, estimates suggest an increase of 35% to 56% will be necessary to satisfy the growing demand by 2050 [2].

Currently, increasing wheat production faces numerous challenges due to biotic and abiotic stressors [3,4]. Among these, salinity is one of the most limiting factors for crop production. Changing climate patterns, unpredictable weather, poor irrigation practices, and the overuse of fertilizers have indeed contributed to elevated salt levels in many salt agricultural areas [5,6]. High soil salinity levels negatively affect crop plants by altering their physiology and metabolism, resulting in adverse impacts on seed germination, seedling growth, development, vegetative and flowering stage growth, fruit setting, and the structural development of the root system [7,8]. Generally, the two major salt-induced effects on plants exposed to salinity are osmotic stress and ionic stress [9,10]. The three main mechanisms for tolerance are osmotic adjustment, ion exclusion and tissue tolerance [3]. Thus, enhancing salt tolerance in crops can be achieved by focusing on key mechanisms that interfere with osmotic adjustment, ion exclusion, i.e., the content of a compatible solute, and the concentrations of Na+ and K+ in plant tissues, in addition to relevant morphological and physiological characteristics.

It is also known that to address soil salinization, the adoption of salt-tolerant genotypes presents a more sustainable and efficient approach compared to other inefficient agricultural practices like leaching and drainage [11]. In this context, robust selection and breeding programs play a crucial role in developing salt-tolerant wheat cultivars. For this purpose, the utilization of local wheat landraces in breeding programs holds significance for developing varieties adapted to specific agro-climatic conditions. In recent years, some researchers have turned their attention to the wheat landraces cultivated in the Saharan oases of Algeria [12,13]. A recent study by [14] revealed the existence of significant variation in salt tolerance in the Algerian oasis wheat landraces, which encourages exploiting them in breeding programs.

The development of salt-tolerant cultivars requires the existence of genetic diversity for the targeted salt tolerance-related trait [15], along with an understanding of the mechanisms governing its inheritance [16]. Systematic crossing using appropriate mating design facilitates the estimation of quantitative genetic parameters like combining ability. For instance, the line × tester mating design is one of the genetic–statistical methodologies that aids in the selection of parental candidates, considering their combining capacity and ability to generate advantageous segregating populations [17]. Combining ability analysis is a crucial genetic tool to evaluate the performance of parental lines and their hybrid offspring. The estimates general combining ability (GCA) measure a parent’s capacity to transmit desirable traits to its progeny, while specific combining ability (SCA) measures the performance of specific hybrid combinations. This analysis guides the selection of superior parents and cross-combinations in breeding programs [18,19].

Previous studies have indicated that certain oasis bread-wheat landraces possess the ability to grow under Saharan conditions, characterized by high temperatures and elevated salt concentrations in irrigation water [12,13]. Therefore, we hypothesize that these wheat landraces could serve as valuable sources for salt-tolerance breeding programs. The objectives of this study were to (1) incorporate selected oasis bread-wheat landraces into a line × tester mating design with other local commercial varieties; (2) evaluate the resulting F2 hybrids for salt tolerance using morphological, physiological, and biochemical traits; and (3) examine how salt stress influenced specific combining ability (SCA) and general combining ability (GCA) for the traits under study.

## 2. Results

### 2.1. Analysis of Variance

A combined ANOVA was conducted to evaluate the effects of genotype, salinity treatment and their interaction on the studied traits (Table 1). The results showed very significant effects of genotype and salinity for all the studied traits (*p* < 0.001). The interaction between genotype and salinity was also very significant (*p* < 0.001) for all traits except for shoot length (SL). The largest genotype mean square was observed for proline (Pro), whereas the mean square for salinity was notably large for Pro, chlorophyll content (Chl), carotenoids content (Cart), shoot fresh weight (SFW) and root fresh weight (RFW).

The ANOVA for the line × tester mating design under control and salt treatments (Supplementary Tables S1 and S2) indicated significant differences (*p* < 0.01) among the genotypes (crosses and parents) for all the traits under investigation, except for shoot length (SL), which showed no significant difference between genotypes and between parents under salt conditions. Parents showed a significant effect for all traits except for (Cart) under control and SL under stress. However, the partitioning of the parental effects revealed a significant effect of lines and testers (each alone) only for RFW and root length (RL) under the control condition, while under salt treatment, no significant effect of lines and testers was observed for all traits.

Further to this, the effect of the interaction between lines and testers (L × T) was noted to be significant for all traits (*p* < 0.05) except SFW, RFW, shoot dry weight (SDW) under control, and SL and sodium ions content (Na^+^) under salt conditions. Additionally, significant differences between crosses in all the studied traits were registered under control and salt conditions (*p* < 0.05), except in SDW under control and SL under salt stress. The mean squares of parents versus crosses were very significant for root dry weight (RDW), Pro, soluble sugars content (Sug), Chl, Cart, Na^+^, potassium ions content (K^+^), and K^+^/Na^+^ ratio under control. Under salt stress, significant differences were registered for SFW, RFW, SDW, relative water content (RWC), Pro, Sug, Chl, Cart, Na^+^, K^+^, and K^+^/Na^+^. The differences between parents and their respective crosses in seedling traits were made clear when exposed to salinity.

### 2.2. Effect of Salinity on Pre-Seedling Growth Traits

The results of the percentage change for SFW, SDW, RFW and RDW are presented in Table 2. Mean values indicated that exposure to salt stress had an adverse impact on the growth parameters of pre-seedlings in hybrids and parents. A significant decrease in SFW and RFW was registered in all genotypes. The reduction in SFW in parents ranged from 25.64% in T4 to 68.96% in L2, while in hybrids it ranged from 34.11% (H9) to 60.72% (H4). The reduction of RFW ranged from 18.46% in T5 to 62.44% in L1 for parents, and from 37.5% in H1 to 47.7% in H4 for hybrids. None of the F2 hybrids outperformed the best-performing parent in terms of SFW; instead, most exhibited a higher reduction when compared to the superior parent.

For SDW of parents, L1, T1 and T5 experienced a minimal reduction of 6% and 7%, respectively, while L2 registered the highest reduction of 75.7% as compared to the control. For hybrids, the reduction in SDW ranged from 7.4% in H9 to 37.5% in H7. The reduction in SDW was statistically non-significant in 13 genotypes (*p* > 0.05). For SDW, no hybrid surpassed the performance of the superior parent, although three hybrids demonstrated close performance to it (H3, H9 and H10).

For RFW, among parents, T5 showed the minimum reduction (10.96%, *p* > 0.05) while L1 revealed the highest reduction (62.44%). Among the hybrids, the reduction in RFW ranged from 37.50% (H1) to 76.75% (H4). No hybrid performed better than the best-performing parent (T5); instead, most of them showed a poor to moderate response in terms of RFW.

The percentage decrease in RDW varied between 4.50% in H7 to 50.60 in H2% for hybrids. For parents, it ranged from 5.7% in T4 to 32.6% in T3. T5 and H1 registered a non-significant increase in RDW. The results of the LSD test revealed that the changes in RDW for T1, T4, T5, L2, H1, H7 and H9 were not statistically significant (*p* > 0.05). Additionally, no hybrid surpassed the superior parent for this trait. Nevertheless, hybrid H1 outperformed all the other testers and lines.

SL exhibited a minimal reduction of 44.97% in T5 and aa maximal reduction of 58.15% in L2 among parents, while a minimal reduction at 40.38% (H10) and a maximal reduction at 56.67% (H7) was recorded for hybrids (Table 2). The decline in RL ranged from 50.34% (T5) to 78.55% (T4) in parents, and from 54.55% (H7) to 86.27% (H5) in hybrids (Table 2). Two hybrids (H10 and H9) surpassed the best parent (T5) in terms of SL, while H1 showed near-parent performance (−45.31%). Moreover, hybrids did not outperform the superior parent for RL (T5). However, H7 and H8 performed better than the remaining parents for the same trait.

Salinity also decreased RWC (Table 2) at a minimum of 2.39% (T2) and a maximum of 11.99% (T5) in parents. Regarding hybrids, RWC exhibited a decline range from 2.90% (H7) to 9.65% (H6). Furthermore, three hybrids (H8, H4, and H1) demonstrated performance intermediate to the best and second-best parents in terms of RWC.

### 2.3. Effect of Salinity on Photosynthetic Pigments

Chl and Cart manifested notable declines due to the detrimental effects of salt stress in all genotypes (Table 3), except for some genotypes that increased Cart under salt stress, namely T3 and its relative hybrid with L2 (H8), which increased Cart by 140% and 61% for T3 and H8, respectively. Salinity led to a marked reduction in Chl that ranged from 47.56% in T4 to 68.03% in T3 for parents (*p* < 0.05), and from 19.38% (H3) to 84.04% (H7) for crosses. On the other hand, for Cart, the maximum decrease among parents was observed in L1 (71.76%), while the minimum decrease was noted in T1 (25.89%). Among hybrids, the most significant decline occurred in H10 (70.014%), whereas H5 recorded the least significant decrease (7%), *p* > 0.05. Additionally, one hybrid registered a lower reduction in Chl as compared to the parent with the most minimal reduction for the same trait. For Cart, all hybrids showed a higher reduction than the best parent (T3).

### 2.4. Effect of Salinity on Compatible Solutes

The result of the effect of salt stress (150 mM) on Pro and Sug is shown in Table 3. Salt stress induced significant enhancements in Pro and Sug in all genotypes, except in tester T3 and its hybrid combination with L1 (H3), which significantly decreased Sug by −27.15% and −60.51% for T3 and H3, respectively. The results of mean values of Pro revealed that a minimum enhancement of 12-fold (T1) and a maximum enhancement of 81-fold (T5) were registered for parents, while in crosses, H9 displayed a 16-fold elevation as the minimum increment, whereas H5 exhibited an impressive 107-fold surge exceeding the value of the parents that experienced the highest increase in Pro. For the Sug among parents, the percentage increase ranged from 40.20% (L1) to 380% (T2), whereas among hybrids, it ranged from 89.93% (H9) to 547% (H10). H10 also outperformed the superior parent that registered the highest increase in Sug.

### 2.5. Effect of Salinity on Ions Content

Salinity imposed a significant accumulation of Na^+^ on seedlings of all genotypes. Consequently, K^+^ was decreased in all genotypes except in testers T4 and T5 and hybrid H5, which increased K^+^ with a percentage increase of 12.75%, 1.96%, and 24.02%, respectively. As a result, the K^+^/Na^+^ ratio was also notably reduced in all genotypes (Table 4). However, the increase in T5 was statistically non-significant (*p* > 0.05). Tester parents remarkably accumulated higher K^+^ in their tissues than line parents.

The percentage increase in Na^+^ ranged from 10-fold (T4 and L2) to 18-fold (T2) in parents, and from 2-fold (H7) to 16-fold (H10) in hybrids. On the other hand, K^+^ fell significantly with a range of 7.28% (T2) to 33.84% (L2) in lines, and from 8.54% (H4) to 37.18% (H2). As a result, the K^+^/Na^+^ ratio also showed a significant drop spanning from 93.48% (T1) to 95.31% (T2) for testers, from 94.28% (L2) to 95.29% (L1) for lines, and from 53.59% (H5) to 95.07% (H10). For Na^+^, 4 hybrids out of 10 (H5, H7, H6 and H8) exceeded the best parent (the parent that accumulated the lowest Na^+^ in its tissues). Additionally, one hybrid (H5) outperformed the superior parent in terms of K^+^. For the K^+^/Na^+^ ratio, two hybrids (H5 and H7) exceeded the best parent by registering the most minimal reduction in the K^+^/Na^+^ ratio.

### 2.6. Correlation and Regression

The correlation analysis (Table 4) showed that SFW had the highest positive correlation coefficient with the total MFVS (r = 0.730), followed by K^+^ (r = 0.674), RDW (r = 0.633) and RFW (r = 0.621). SL and SDW were also significantly and positively correlated to MFVS. A stepwise multiple linear regression model was employed to evaluate the relationship between the total MFVS as the dependent variable and the MFV of the different studied traits (Table 5). The model explains approximately 77.2% (R^2^ = 0.77) of the variation in the total MFVS. SFW emerged as the strongest predictor (B = 0.348, *p* < 0.001), followed by Pro (B = 0.165, *p* < 0.01). The regression equation can be written as MFVS = 0.169 + 0.347 × SFW + 0.164 × Pro.

### 2.7. Effect of Salinity on General Combining Ability (GCA)

The results of general combining ability (GCA) under control and salinity are shown in Table 6 and Table 7. The magnitude of GCA ranged from −229.76 (Pro, T4) to 364.21 (Sug, T3) under control, and from −254.81 (Pro, L1) to 310.88 (Pro, T2). Under control, testers T4 and T5 were the best general combiners for RFW, while T2 was the best combiner for SDW. However, these parents did not show highly significant desirable GCA effects for RFW and SDW when exposed to salinity, and neither of the other parents did. L1 expressed significant positive effects for SL under the control condition, while only T5 had significant GCA effects for SL under salinity. No significant positive effects for RL were registered under control; however, T1 and T3 were good combiners for RL under salt stress.

For RWC, Pro, Sug, and Chl, no parent registered a significant effect of GCA under normal conditions. However, under salinity, L2, T1, and T2 were good combiners for Pro; T2, L1, T1, and T4 emerged as good combiners for Sug; L1, T1 and T5 were good combiners for Chl; and T4 was the only good combiner for RWC. For Cart, four parents expressed significant a GCA effect under control, although these effects were altered by salinity where no parent registered significant GCA effects.

For ionic content, five parents under control and three under stress registered significant negative GCA for Na^+^. From these, L1 and T3 expressed consistent significant desirable effects of GCA for Na^+^ under both conditions. For K^+^, L1, T2 and T3 showed significant positive GCA under control. Under stress, only T2 maintained positive effects for K^+^, although the magnitude was negligible and lacked statistical significance (*p* > 0.05). Furthermore, T3 was a good combiner for K^+^/Na^+^ under both conditions. T4 was the best general combiner for K^+^/Na^+^ under salt stress. The frequency of significant favorable effects of ionic content traits was higher under control conditions than salt conditions.

Under saline conditions, tester T1 emerged as the best combiner, exhibiting significant GCA effects for four traits, including Pro, Sug, Chl, and RL. Conversely, under control conditions, L1, T2, T3 and T5 demonstrated superiority by expressing significant GCA effects for four traits simultaneously: T5 emerged as an excellent combiner for RFW, Cart, Na^+^, and K^+^/Na^+^; T3 showed significantly good combining ability for ionic balance (Na^+^, K^+^ and K^+^/Na^+^) and Cart; T2 was superior for SDW, Cart and ionic balance; and L1 was good combiner for SL, Cart and Na^+^ and K^+^.

### 2.8. Effect of Salinity on Specific Combining Ability (SCA)

**Figure 1:**
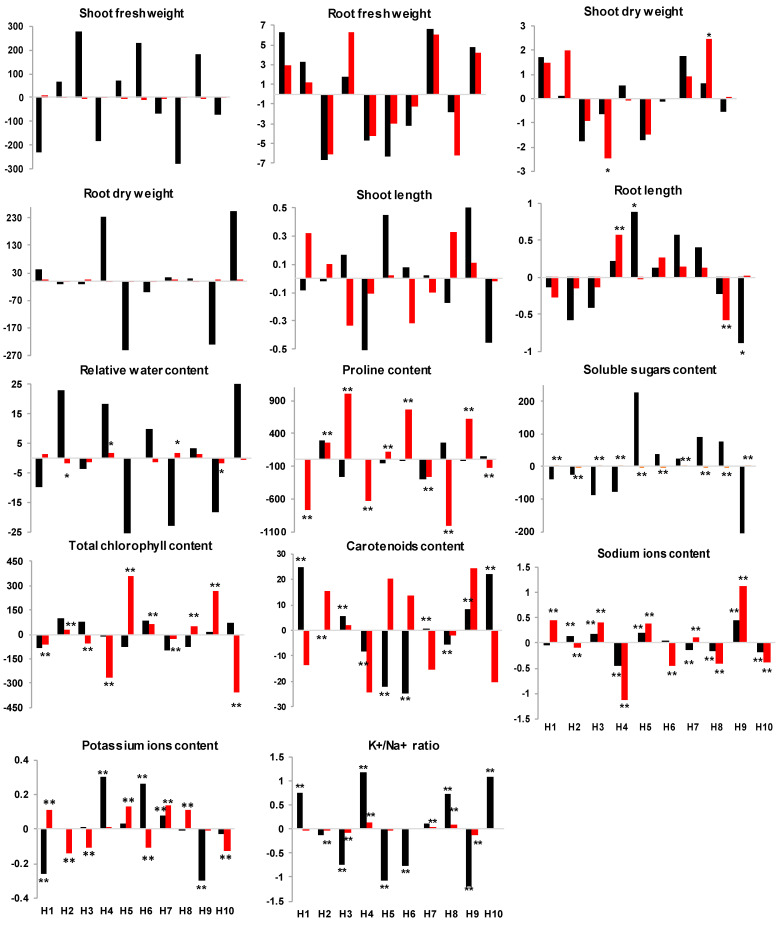
General combining ability effects of studied traits in lines and testers under control and salt-stress conditions. (■), control, (■) salt treatment. *, ** significant at 5% and 1%, respectively, as determined by the *t*-test.

The results shown in Figure 1 reveal that hybrids did not show significantly improved behavior than expected based on the GCA of their parents in terms of SFW, RFW, SDW and RDW under control conditions. H9 behaved significantly better than expected for SDW (*p* < 0.05) under the stress condition. H5 showed significant effects under control for RL, while H4 recorded significant effects under salinity for the same trait. Hybrids did not show significant SCA effects for RWC, Pro, Sug and Chl under normal conditions. On the other hand, under salinity, significant desirable SCA effects for two hybrids in RWC (H4 and H7), five hybrids in Pro (H2, H3, H5, H6 and H9), four hybrids in Sug (H3, H4, H7 and H7), and five hybrids in Chl (H2, H5, H6, H8, H9) were registered. Contrastingly, for Cart, five crosses showed significant positive effects of the SCA under control, while no significant effect was registered under salt stress.

For ionic content, four hybrids (H4, H7, H8 and H10) exhibited significantly better performance than expected, accumulating lower Na^+^ levels than predicted based on the GCA of their parents under control. Under salinity, 50% of hybrids obtained negative significant SCA effects. H4, H8 and H10 showed consistent significant negative SCA effects under both conditions for Na^+^. H4 and H6 recorded significant positive SCA effects for K^+^ under control conditions, while four hybrids (H1, H5, H7 and H8) exhibited desirable significant effects for K^+^ upon exposure to salinity, with H7 displaying consistent significant positive effects for K^+^ under both conditions. For the K^+^/Na^+^ ratio, four favorable significant effects were registered in H1, H4, H8 and H10 under normal conditions, whereas under salinity, only two positive significant effects were recorded in H4 and H8, with hybrid H4 and H8 behaving significantly better than expected for this trait under both conditions.

H4 emerged as an exceptional hybrid under salt stress, showing favorable SCA for Na^+^, K^+^/Na^+^ ratio, RL, RWC, Sug. Under normal conditions, H7 and H10 behaved significantly better than expected for three traits simultaneously: Cart, Na^+^ and K^+^/Na^+^ for H10 and Cart, K^+^ and Na^+^ for H7.

### 2.9. Genetic Component of the Total Variance

The variance due to the SCA was higher than the variance due to GCA, given the value of σ^2^_GCA_/σ^2^_SCA_ that was lower than the unit (1 > σ^2^_GCA_/σ^2^_SCA_) under both normal and salt conditions. These results are substantiated by the ratio σ^2^_A_/σ^2^_D_, which was also lower than the unit in these traits (Table 8).

## 3. Discussion

A key strategy for improving wheat productivity is enhancing the abiotic stress tolerance of wheat varieties [20]. Specifically, developing genotypes with the genetic capacity to thrive under high soil salinity offers a sustainable solution to boosting yields. This approach reduces the reliance on costly and labor-intensive soil remediation techniques. In this context, the present study focuses on evaluating the response of some wheat hybrids and their parents to salt stress at the seedling stage, aiming to determine the nature of gene action (additive and non-additive effects, as assessed through GCA and SCA) for key traits related to salt tolerance. By exploring these genetic factors, the goal is to identify and develop breeding material for salt-stress resistance, ultimately paving the way for higher-yielding, more resilient wheat varieties.

Significant differences among genotypes (parents and their hybrids) were observed for all traits, suggesting the presence of genetic variation in these genotypes. This variation was most pronounced in Pro, indicating substantial genetic variation among genotypes in their ability to accumulate Pro. Additionally, salinity treatment significantly affected all traits, and the effects were more pronounced in Pro, Chl, Cart, SFW and RFW. Furthermore, significant genotype × salinity interactions were found for almost all traits, implying that the genotypes responded differently to salt stress. The significant effects of parents versus crosses means that crossing parents have tapped into wider variability pertaining to the studied traits. The interaction between lines and testers highlights the importance of the genetic backgrounds represented by the lines and testers, as well as their interplay in determining the phenotypic outcomes of most of the studied traits.

The imposed salt stress had adverse effects on pre-seedling development, resulting in marked reductions in both root and shoot biomass accumulation. Moreover, the elongation of shoots and roots was substantially impaired under saline conditions. These observations are consistent with the well-documented inhibitory effects of salt stress on early plant growth in wheat due to osmotic stress and ion toxicity [19,21,22,23,24]. The accumulation of salt in the growing medium limits water uptake and causes physiological drought and the over-accumulation of salt ions in plant tissues, which results in ion toxicity that impede growth processes, resulting in restricted development under saline conditions [25]. As a result, growth inhibition occurs due to reduced leaf initiation and expansion, stunted internode development, and accelerated leaf shedding [26]. In the current study, 7 genotypes out of 17 displayed non significance changes in RDW. Moreover, SDW was reduced in all genotypes, although the reduction was not statistically significant in 13 genotypes. In fact, shoot development has been observed to exhibit greater sensitivity to salt stress compared to root growth. By limiting leaf growth while maintaining root development, plants can reduce water loss and prevent salt buildup in their environment [27].

Salt stress induced a significant accumulation of Pro and Sug. Osmoregulation is primarily achieved by accumulating organic and inorganic solutes like proline and total soluble sugars during salinity, to lower the water potential without reducing the actual water content [26]. In addition to their role in osmotic adjustment, compatible solutes contribute to stabilizing subcellular structures such as membranes and proteins, scavenging free radicals, and maintaining cellular redox balance during stressful conditions [28].

The results demonstrated that salt stress induced a significant reduction in the Chl across all genotypes. Conversely, the response of Cart to salinity was more variable among the studied genotypes. While most genotypes exhibited a significant decrease in Cart levels under salt stress, genotype T3 showed a significant increase.

Additionally, genotypes L2 and H5 displayed a statistically non-significant reduction; this result is in line with that of Pastuszak et al. [29], where a salt-tolerant line increased Cart by 11% under 150 mM salt stress. The accumulation of Cart in this genotype under salt stress may play a crucial role in maintaining photosynthetic efficiency and protecting chloroplasts from oxidative damage.

Furthermore, salinity caused a significant accumulation in Na^+^ compared to control; accordingly, K^+^ was reduced, except for in T5, which showed no significant difference between control and treatment, and in T4 and H5, which registered a slight significant increase. Similar results were also verified by other researchers [14]. According to Shabala and Pottosin [30], salt-resistant varieties potentially possess advanced potassium homeostasis mechanisms with two-pore K^+^ channels and shakertypes, non-selective cation channels that aid in the permeability of K^+^ and transporters (HKT, KUP/HAK/KT and K^+^/H^+^). Because of Na^+^ accumulation and K^+^ reduction under salt stress, the K^+^/Na^+^ ratio was significantly reduced in all genotypes. However, hybrid H5 demonstrated an exceptional response, maintaining a stable K^+^/Na^+^ ratio without significant alterations in ion discrimination. This unique behavior of H5 may be attributed to its capacity to sustain, or potentially increase, cytoplasmic K^+^ levels in shoot tissues under saline conditions. Such a mechanism could contribute to the maintenance of cellular homeostasis and potentially enhance salt tolerance in this genotype.

The strongest positive and significant correlation was observed between SFW and MFVS. This indicates that wheat genotypes with greater SFW exhibit higher salt tolerance at the seedling stage. The other pre-seedling traits, like SL, RFW and RDW and K^+^, also showed significant positive associations with MFVS. The stepwise multiple regression model identified two key predictors: SFW and Pro. Despite Pro’s weak correlation with the total MFVS (r = 0.102), it emerged as a significant predictor in the multiple regression model. This suggests that Pro’s role in salt tolerance at the seedling stage becomes more apparent when analyzed alongside other physiological parameters, particularly SFW. In addition, the high R^2^ value of the regression model indicates that Pro’s impact on salt tolerance is driven by complex interactions, rather than being directly linked in a linear fashion. SFW was the most important trait for predicting salt tolerance, as evidenced by its highest correlation with MFVS and its dominant role in the regression model. Thus, it should be considered a primary selection trait.

The evaluation of breeding material fundamentally relies on analyzing genetic variation and assessing the mean performance of parents and their hybrid progeny [31]. In most self-pollinating crops like wheat, breeding involves crossing parent lines and then selecting the best offspring. If no descendants were better than their parents, plant breeding would not be effective [32]. The assessment of F2 populations in wheat breeding is particularly valuable as it represents the first generation where genetic segregation occurs, allowing breeders to identify promising combinations for further selection. In the current study, the results revealed the existence of variation among the F2 hybrids’ performance under salt stress, which indicates good potential for selection in segregating generations. Furthermore, the existence of hybrids that surpassed the performance of the superior parent indicates the existence of significant transgressive segregation across the different studied traits. Notably, four hybrids demonstrated lower Na^+^ accumulation compared to their parents, indicating enhanced salt-exclusion capacity. Hybrid H5 emerged as the most promising hybrid, showing exceptional performance across multiple traits, including Pro accumulation, Na^+^ exclusion, K^+^ uptake, and K^+^/Na^+^ discrimination. Various factors have been proposed to explain the emergence of transgressive phenotypes in segregating populations, such as high mutation rates in hybrids, instability in development, non-additive or epistatic gene interactions, unmasking recessive alleles, chromosome number variations, and complementary actions of additive alleles from the parental lines [33].

Knowledge of the genetic basis and inheritance of salt-tolerance traits is essential for breeders to develop effective strategies in creating salt-tolerant crop varieties [34]. Combining ability is an essential tool in plant breeding for selecting parents and segregating populations [35]. While additive gene effects are responsible for GCA, non-additive gene effects are responsible for SCA [36].

GCA is determined by the average performance of a genotype across a set of crosses involving different parents. Therefore, selecting high-performing parents based on their GCA effects is essential for successful breeding [36]. The results revealed that parent T3 exhibited superior GCA for both Na^+^ exclusion and K^+^/Na^+^ ratio maintenance across stress and control conditions, indicating its potential value in breeding programs for salt tolerance. L1, T2, T3 and T5 were good combiners for four traits simultaneously under control, and most of them were related to ion homeostasis. However, under salinity, T1 was a good combiner for four traits mainly related to compatible solutes. Parents exhibiting high and significant GCA for traits could be valuable in developing salt-tolerant varieties, achieved through a process of hybridization followed by selective breeding techniques. Performing a mating design analysis (such as line × tester and diallel) across different environments helps identify the combining ability × environment interaction, making the process more efficient by providing environment-specific recommendations for the best parents and populations [37].

Typically, promising crosses are chosen based on SCA, which reflects the deviation from the predicted performance derived from the GCA of their parents. Selecting crosses solely based on their individual performance is not effective. Therefore, genetically superior crosses should also exhibit positive and significant SCA effects for the traits being studied [38]. Most hybrids exhibited significant SCA effects under salt stress for three traits—predominantly physiological and biochemical traits, rather than pre-seedling growth traits. Moreover, the analysis revealed notable consistency across environmental conditions for certain ionic homeostasis parameters. Specifically, three hybrid combinations exhibited stable SCA effects for Na^+^ concentration under both non-saline and saline conditions. Additionally, two distinct hybrids demonstrated consistent SCA effects for the K^+^/Na^+^ ratio across both environments. One hybrid was noted to have a consistent SCA effect for K^+^ under both conditions. This suggests that these hybrids might have a stable genetic mechanism for regulating Na^+^ exclusion, K^+^ uptake and K^+^/Na^+^ discrimination and that the non-additive gene effects governing these traits are expressed similarly under different environmental conditions. Thus, their consistent performance suggests that their behavior under salt stress could be more predictable, making selection and advancement in breeding programs more efficient.

Under both conditions, most hybrids with significant SCA were derived from crosses between parents with contrasting GCA status, specifically good × poor or poor × good combiners. In contrast, a smaller proportion of hybrids with significant SCA originated from parents with SCA status. Under control conditions, only 16% and 12.5% of hybrids with significant SCA resulted from poor × poor and good × good parental combinations, respectively. Similarly, under saline conditions, 20.64% and 24.13% of hybrids with significant SCA were derived from poor × poor and good × good combiner crosses, respectively. This result implies that predicting hybrid performance based solely on parental GCA may not be reliable, and the extensive testing of hybrid combinations is necessary. Hence, a breeding strategy focusing on specific hybrid combinations, rather than just parental line performance, could be most effective. The line × tester analysis unveiled the predominance of non-additive gene effects in controlling all the traits studied under both conditions—similar findings were obtained by Zafar et al. [39]. Contrary to these results, Mohammadi et al. [40] observed the predominance of an additive effect that seems to govern ion hemostasis-related traits in rice hybrids under salt stress. This suggests that the effective selection for the salt tolerance-related traits studied should begin in later generations [40]. However, superior gene combinations can still be fixed through careful selection, and some transgressive segregants may be stabilized in later generations.

## 4. Methods

### 4.1. Plant Material

Plant material consisted of five locally sourced bread-wheat landraces collected from two oases from the Algerian Sahara (Touat and Oued Righ). These landraces were designated as testers [14]. In addition, 2 local commercial varieties were used as lines along with 10 cross-combinations (F2) that resulted from crossing parents, following a line × tester mating design during the 2021/2022 growing season; then, the selfing of F1 hybrids was conducted during the next growing season (2022/2023). The parents’ names, sources and code along with their relative hybrids are presented in Table 9.

### 4.2. Experimental Design

The study was conducted during 2023/2024 (December–January) at Chaabat Erssas, University of Mentouri’s Brothers, Constantine 1, Algeria (36°20′24.0″ N; 6°37′12.0″ E). Seeds were surface sterilized with 5% sodium hypochlorite (NaOCl) solution for 5 min, followed by thorough rinsing with distilled water. Subsequently, seeds were imbibed in distilled water for 24 h. Uniform seeds were then germinated in Petri dishes (25 seeds per dish, three replications) on filter paper moistened with either 10 mL of distilled water (control) or 10 mL of 150 mM NaCl solution (salinity treatment). To maintain moisture, 5 mL of the respective solution was added every 48 h. Germination was carried out at an ambient temperature of 17 ± 3 °C. After 10 days, seedling growth parameters were measured.

The remaining seedlings in Petri dishes were transferred into small plastic containers of 180 mL (diameter = 7 cm) with drainage holes. They were filled with a mixture of peat and sable (2:1) with a rate of three plants per container. The plants were irrigated with nutrient solution prepared by dissolving hydrofer fertilizer (20% N, 20% P_2_O_5_, 20% K_2_O, 0.10% Fe, 0.10% MgO, 0.05% B, 0.02% Mn, 0.01% Cu, 0.01% Zn) in distillated water for the control and saline solution (150 mM of NaCl) for the treatment. The pH of the nutrient solution was adjusted using acid and base (KhPO_4_ and Kh_2_PO_4_) and was maintained at 5–5.5 (JENWAY pH meter 3520). Irrigation was performed every 48 h with 90 mL of the respective treatment solutions. This approach mimics field conditions in water-limited environments, where plants receive restricted, periodic water inputs instead of continuous or complete water deprivation, as recommended by Tavakkoli et al. [41].

The electrical conductivity (EC) of the drained solution was monitored (JENWAY conductimeter 4510) to maintain consistent salinity stress. When the EC exceeded the target value of approximately 13.3 dS·m⁻^1^, containers were flushed with distilled water to prevent salt accumulation in the growth medium.

The experiment was conducted in a glasshouse under an open natural environment, using a randomized complete block design with five replications. The glasshouse environment was characterized by a temperature range of 2–21 °C, an 11 h photo period, and a relative humidity of 50 ± 7%. After three weeks of salinity treatment, seedlings were harvested for physiological and biochemical analyses.

### 4.3. Data Collection

At the germination stage (10 days of germination), the pre-seedlings were assessed for shoot fresh weight (SFW), root fresh weight (RFW), shoot dry weight (SDW), root dry weight (RDW), shoot length (SL), root length (RL), and relative water content (RWC) according to Barrs and Weatherley [42]. Fresh leaves were weighed, saturated in in distillated water for 24 h, and dried to constant mass. The resulting fresh, turgid, and dry weights were used to compute RWC using a standard formula: RWC % = (FW − DW)/(TW − DW) × 100. After extended exposure to salinity, seedlings were harvested to assess the following traits.

#### 4.3.1. Total Chlorophyll and Carotenoids

Fresh leaves (100 mg) were immersed and punched in 10 mL 80% acetone. After that, they were incubated in the dark for 48 h. Absorbance readings were taken at 663 nm, 646 nm, and 470 nm. Total chlorophyll (Chl) and carotenoid (Cart) pigments were calculated as follows [43]: $C_{a}=12.21A_{663}-2.81A_{646}$; $C_{b}=20.13A_{646}-5.03A_{663}$; $A_{c+x}=(1000A_{480}-1.29C_{a}-81.4C_{b})/227$.

#### 4.3.2. Proline

Fresh leaves (500 mg) were homogenized in 10 mL of 3% sulfosalicylic acid solution on ice. After centrifugation (SIGMA 2-6E centrifuge), 2 mL of the supernatant was combined with 2 mL of acid-ninhydrin and 2 mL of glacial acetic acid. The reaction mixture was incubated in a water bath for 1 h and then cooled in an ice bath. Toluene (4 mL) was added to each tube and vigorously vortexed. The absorbance of the upper phase was measured at 520 nm using a spectrophotometer (JENWAY 6700 series). Proline (Pro) concentration was determined using a standard curve prepared with L-proline [44].

#### 4.3.3. Total Soluble Sugars

Fresh leaves (100 mg) were ground in 3 mL of 80% ethanol and kept in darkness for 48 h. The resulting extract was evaporated under a stream of hot air, and the residue was dissolved in 20 mL of distilled water. A 2 mL portion of the diluted extract was combined with 1 mL of 5% phenol and 5 mL of sulfuric acid. After incubating the mixture at room temperature for 20 min, the absorbance was measured at 485 nm [45]. Total soluble sugars (Sug) were quantified using a standard curve prepared with glucose.

#### 4.3.4. Ions Content

The sodium ion content (Na^+^) and potassium ion content (K^+^) in dried seedlings’ shoots were determined according to Asch et al. [46]. Seedlings were rinsed with distilled water and then dried in an oven at 80 °C. Leaf powder (100 mg) was digested in 10 mL of 1 N hydrochloric acid overnight with mechanical agitation at room temperature. The solution was filtered into a 100 mL flask and diluted to 100 mL with distilled water. Subsequently, the samples were analyzed using a flame photometer (JENWAY PFP7), and the concentrations of Na^+^ and K^+^ were determined using standard curves prepared with KCl and NaCl.

### 4.4. Statistical Analysis

The collected data were analyzed to determine descriptive statistics (mean and standard deviation), graphs, and Fisher’s least significant difference (LSD) at 5% using XLSTAT 2016 V1.0 (v18) statistical package (XLSTAT Addinsoft Inc., New York, NY, USA). The salt-tolerance coefficient for each genotype was calculated using the following formula [47]:

$$STC=\frac{Value\;for\;salt\;treatment}{Value\;for\;control\;ctreatment}\times 100$$

The computed STC was used to determine the membership function value of each trait for all genotype using the following equations [48]:

$$MFV1=\frac{{STC}_{i}-{STC}_{min}}{{STC}_{max}-{STC}_{min}}\;MFV2=1-\frac{{STC}_{i}-{STC}_{min}}{{STC}_{max}-{STC}_{min}}$$

where STC_i_ is the salt-tolerance coefficient of a given genotype and STC_min_ and STC_max_ are the minimum and maximum salt-tolerance coefficient amongst all genotypes. The calculated MFVs of all the studied traits were averaged for each genotype to obtain values between zero and one, to rank genotypes from most tolerant to least tolerant. Pearson’s correlation coefficient was used to explore the association between the total MFVs and the MFVs of different traits. Stepwise multiple linear regression was conducted using the total MFVS as dependent variable and the MFVs of different traits as independent variables. RStudio 2023.09.1-494 (Posit PBC, Boston, MA, USA), package “Agricolae,” was used to conduct a line × tester analysis, as suggested by Kempthorne [49] and applied by Singh and Chaudhary [50], including a line × tester ANOVA, the general combining ability (GCA) effects of lines and testers, and the specific combining ability (SCA) effects of hybrids. The significance of GCA and SCA effects was tested using the *t*-test.

## 5. Conclusions

This study aimed to evaluate the effects of salt stress (150 mM) on the performance and combining ability of 10 F_2_ hybrids and their parents, developed through a line × tester design. The findings revealed notable genetic variation among these genotypes, as demonstrated by their distinct responses to 150 mM salinity stress. The hybridization process facilitated the development of transgressive segregants with improved performance in certain traits, particularly those related to ion homeostasis. Among the genotypes tested, SFW emerged as the strongest predictor of salt tolerance, demonstrating the highest correlation with MFVS and the greatest contribution in the regression model, highlighting its critical role in selecting salt-tolerant genotypes under the specific conditions of this study. The observed variation in the studied traits was predominantly governed by non-additive genetic effects. H5 exhibited superior performance, surpassing the best parent for Na⁺, K⁺, K⁺/Na⁺, and Pro. Additionally, T1 was identified as a good combiner for Pro, Sug, Chl, and RL under salinity stress. In contrast, under control conditions, L1, T2, T3, and T5 exhibited high GCA effects across four traits simultaneously. H4 demonstrated excellent potential under salt stress, with significant SCA effects for Na⁺, K⁺/Na⁺ ratio, RL, RWC, and Sug, while under control conditions, H7 and H10 performed significantly better than expected for three traits. Given that non-additive genetic effects predominated across all studied traits, selection at early generations may be less effective, and breeding strategies should prioritize later-generation selection when genetic recombination has stabilized. These parental and hybrid combinations provide insight into their potential use in breeding programs targeting salt tolerance under the tested conditions.

## Figures and Tables

**Table 1:** General linear model ANOVA for hybrids and their parents under control and salt-stress conditions.

	Genotype	Salinity	Genotype × Salinity	Error
SFW	2118.38 ***	163,348.03 ***	984.89 ***	378.46
RFW	290.70 ***	29,902.47 ***	330.36 ***	52.32
SDW	27.57 ***	119.16 ***	16.05 ***	4.83
RDW	3.054 ***	57.15 ***	3.36 ***	0.54
SL	1.756 ***	436.68 ***	0.49	0.37
RL	2.181 ***	302.11 ***	1.52 ***	0.29
RWC	5.291 ***	1125.36 ***	8.82 ***	0.62
Pro	1.1 × 10^6^ ***	3.1 × 10^8^ ***	1.2 × 10^6^ ***	585.65
sug	254.82 ***	995.95 ***	120.80 ***	0.28
Chl	150,611.68 ***	2.2 × 10^7^ ***	1.3 × 10^5^ ***	4441.00
Cart	4277.09 ***	28,231.18 ***	5274.34 ***	21.00
Na^+^	1.742 ***	573.92 ***	2.15 ***	0.02
K^+^	0.073 ***	2.59 ***	0.17 ***	0.005
Na^+^/K^+^	3.817 ***	477.31 ***	3.92 ***	0.08

*** significant at 0.1%, as determined by the F-test.

**Table 2:** Percentage change (%) in pre-seedling growth-related parameters of parents and hybrids.

	SFW	RFW	SDW	RDW	SL	RL	RWC %
T1	−35.9 ± 6.2 ^de^	−56.6 ± 11.5 ^abc^	−7.1 ± 7.2 ^cd^	−16.7 ± 27.3 ^bc^	−50.3 ± 12.8 ^ab^	−69.9 ± 3.1 ^bcde^	−7.4 ± 0.4 ^defgh^
T2	−44.2 ± 12.2 ^bcd^	−32.4 ± 20.9 ^de^	−33 ± 4.7 ^abc^	−26.1 ± 30.2 ^abc^	−49.4 ± 15.6 ^ab^	−58.9 ± 3.1 ^ef^	−2.4 ± 1.3 ^k^
T3	−43.4 ± 0.5 ^bcd^	−55.2 ± 6.2 ^abc^	−10 ± 0.6 ^bcd^	−32.6 ± 14.8 ^abc^	−50.6 ± 12.9 ^ab^	−73 ± 19.2 ^abcd^	−6.3 ± 0.3 ^ghi^
T4	−25.6 ± 27.3 ^e^	−29.1 ± 25.9 ^de^	−8.1 ± 16.1 ^cd^	−5.8 ± 32.9 ^cd^	−45.3 ± 2.8 ^ab^	−78.5 ± 2.6 ^ab^	−5.6 ± 0.4 ^hi^
T5	−44.1 ± 13.3 ^bcd^	−10.9 ± 26.1 ^e^	−7.5 ± 7 ^cd^	40.9 ± 15.6 ^e^	−44.9 ± 15.1 ^ab^	−50.3 ± 1.1 ^f^	−12 ± 0 ^a^
L1	−45.7 ± 24.9 ^bcd^	−62.4 ± 2.3 ^ab^	−6.3 ± 25.1 ^d^	−9.8 ± 5.6 ^bc^	−48.0 ± 15.4 ^ab^	−77.4 ± 12.3 ^abc^	−10.9 ± 0.7 ^ab^
L2	−68.9 ± 4.6 ^a^	−58.6 ± 10.8 ^abc^	−47.8 ± 0.8 ^a^	−32 ± 21.4 ^abc^	−58.1 ± 6.8 ^a^	−69.4 ± 12.3 ^bcde^	−7.9 ± 2.3 ^cdefg^
H1	−37.4 ± 2.2 ^cde^	−37.5 ± 19.7 ^cd^	−13 ± 0.9 ^bcd^	26.7 ± 31.1 ^de^	−45.3 ± 3.3 ^a^	−61 ± 6.6 ^def^	−4.8 ± 0.5 ^ij^
H2	−54.4 ± 4.6 ^abcd^	−59.6 ± 0.6 ^ab^	−17 ± 10.8 ^bcd^	−50.6 ± 2.4 ^a^	−56.2 ± 1 ^a^	−64.4 ± 3 ^cdef^	−8.7 ± 1.8 ^cdef^
H3	−37.4 ± 9.8 ^cde^	−63 ± 8.5 ^ab^	−8.3 ± 29.8 ^d^	−26.3 ± 24.9 ^abc^	−47.2 ± 11 ^ab^	−73.1 ± 6.4 ^abcd^	−6.8 ± 1.7 ^fghi^
H4	−60.7 ± 13.8 ^ab^	−76.7 ± 8.6 ^a^	−33.2 ± 28 ^abc^	−41.2 ± 23 ^ab^	−52.9 ± 14.7 ^ab^	−76.5 ± 7.1 ^abc^	−7.2 ± 1 ^efgh^
H5	−53.4 ± 5.1 ^abcd^	−73.5 ± 2.5 ^a^	−15.4 ± 9.1 ^bcd^	−32.2 ± 2.2 ^abc^	−53.9 ± 4.7 ^ab^	−86.2 ± 6.4 ^a^	−8.7 ± 0.2 ^cdef^
H6	−53.7 ± 17.3 ^abcd^	−66.3 ± 20.2 ^ab^	−13.4 ± 26.2 ^cd^	−37 ± 6.6 ^ab^	−55.3 ± 2.5 ^a^	−75.9 ± 1.6 ^abc^	−9.7 ± 0.6 ^bc^
H7	−50.2 ± 11 ^abcd^	−46.4 ± 13.8 ^bcd^	−37.5 ± 2.1 ^ab^	−4.5 ± 36 ^cd^	−53.3 ± 7 ^ab^	−56.1 ± 3.7 ^f^	−2.9 ± 0.7 ^jk^
H8	−53.5 ± 10.9 ^abcd^	−67.7 ± 1.2 ^ab^	−16.4 ± 3.9 ^bcd^	−43 ± 7.8 ^ab^	−56.6 ± 7.9 ^a^	−54.5 ± 19.1 ^f^	−9.2 ± 2.6 ^bcde^
H9	−34.1 ± 2.9 ^de^	−54.9 ± 2.2 ^abc^	−7.5 ± 34.2 ^d^	−21.6 ± 14.9 ^abc^	−43.5 ± 8.1 ^ab^	−63.9 ± 14.1 ^cdef^	−4.9 ± 0.4 ^ij^
H10	−56.2 ± 1.9 ^abc^	−66.4 ± 1.5 ^ab^	−9.5 ± 15.6 ^bcd^	−42.3 ± 1.4 ^ab^	−40.3 ± 12.1 ^b^	−63.4 ± 8.5 ^cdef^	−9.5 ± 2.4 ^bcd^

Different lowercase letters indicate significant difference according to Fishers LSD test at 5%.

**Table 3:** Percentage change (%) in physiological traits of parents and hybrids’ seedlings.

	Pro	Sug	Chl	Cart	Na^+^	K^+^	K ^+^/Na^+^
T1	12.5 ± 17.9 ^a^	374.5 ± 57.3 ^i^	−65.4 ± 0.2 ^b^	−25.9 ± 0.3 ^a^	12.7 ± 53.2 ^f^	−10.9 ± 2.7 ^e^	−93.5 ± 0.3 ^abc^
T2	34.1 ± 55.1 ^d^	380 ± 93.6 ^i^	−67.6 ± 0.2 ^g^	−49.9 ± 1.3 ^cde^	18.7 ± 90.9 ^i^	−7.3 ± 9.1 ^e^	−95.3 ± 0.6 ^a^
T3	27.9 ± 80 ^bcd^	−27.2 ± 3.3 ^ab^	−68 ± 0.1 ^e^	140.7 ± 8.1 ^f^	15.5 ± 65.8 ^g^	−10.5 ± 11.5 ^e^	−94.6 ± 0.9 ^ab^
T4	18.5 ± 31.9 ^abc^	205.7 ± 38.7 ^h^	−47.6 ± 0.1 ^f^	−44 ± 0.4 ^ab^	10.7 ± 171.2 ^e^	12.8 ± 7 ^g^	−90.4 ± 0.8 ^d^
T5	81.3 ± 158.4 ^f^	78.4 ± 15.4 ^cd^	−55.2 ± 1.4 ^i^	−68.1 ± 1.9 ^cde^	17.5 ± 163.5 ^hi^	2 ± 3 ^f^	−94.5 ± 0.7 ^ab^
L1	27.8 ± 149.2 ^bcd^	40.2 ± 4.3 ^bc^	−61.4 ± 0.2 ^h^	−71.8 ± 0.2 ^a^	17.2 ± 64.2 ^ghi^	−14.4 ± 3.7 ^de^	−95.3 ± 0.3 ^a^
L2	80.7 ± 121.9 ^f^	98.1 ± 14.5 ^cdef^	−61.8 ± 0.1 ^c^	−7.6 ± 0.8 ^bc^	10.7 ± 246.7 ^e^	−33.8 ± 5.9 ^ab^	−94.3 ± 1.7 ^ab^
H1	20.1 ± 79 ^abc^	130.5 ± 7.9 ^defg^	−61.9 ± 0 ^d^	−49.1 ± 0.2 ^a^	13.2 ± 117 ^f^	−14.1 ± 2.6 ^de^	−94 ± 0.7 ^ab^
H2	29.3 ± 72.2 ^cd^	155.6 ± 31.7 ^efgh^	−74.6 ± 0 ^d^	−34.8 ± 1.7 ^cde^	7.7 ± 17.4 ^bc^	−37.2 ± 3.8 ^a^	−92.8 ± 0.6 ^abcd^
H3	62.5 ± 152.6 ^e^	−60.5 ± 2.6 ^a^	−19.4 ± 0.1 ^e^	−20.9 ± 0.1 ^a^	10 ± 101.1 ^de^	−21.1 ± 6.1 ^cd^	−92.9 ± 1.2 ^abcd^
H4	23.2 ± 24.3 ^abcd^	196.1 ± 15 ^gh^	−81.9 ± 0.1 ^e^	−54.4 ± 3.6 ^e^	9 ± 68.6 ^cd^	−8.5 ± 5.3 ^e^	−90.9 ± 1.1 ^d^
H5	107.1 ± 163.1 ^h^	114.6 ± 12.2 ^cdef^	−59.2 ± 0 ^d^	−7 ± 0.7 ^bcd^	1.7 ± 12.3 ^a^	24 ± 7.1 ^h^	−53.6 ± 4.8 ^f^
H6	63.9 ± 442 ^e^	366.1 ± 53.6 ^i^	−74.1 ± 0 ^d^	−28.9 ± 2 ^e^	6.8 ± 36.2 ^b^	−31.4 ± 1.3 ^ab^	−91.2 ± 0.4 ^cd^
H7	18.1 ± 17 ^ab^	166.9 ± 24.7 ^fgh^	−84 ± 0.1 ^c^	−50.5 ± 2.7 ^cde^	2.3 ± 37 ^a^	−26.8 ± 2.2 ^bc^	−77.8 ± 3 ^e^
H8	32.8 ± 39.4 ^d^	121.3 ± 23.9 ^defg^	−64.4 ± 0.2 ^a^	61.3 ± 0.7 ^bc^	7 ± 67 ^b^	−27.3 ± 4.3 ^bc^	−91 ± 0.5 ^cd^
H9	16.6 ± 4.1 ^a^	89.9 ± 14.4 ^cde^	−72.2 ± 0 ^d^	−26.2 ± 1.7 ^cde^	10.2 ± 54.8 ^de^	−12.1 ± 5.3 ^de^	−92.1 ± 0.8 ^bcd^
H10	60.6 ± 178.6 ^g^	548 ± 125.1 ^j^	−83.6 ± 0.1 ^f^	−70 ± 3.3 ^de^	16.3 ± 96.5 ^gh^	−14.8 ± 5.4 ^de^	−95.1 ± 0.4 ^a^

Different lowercase letters indicate significant difference according to Fishers LSD test at 5%.

**Table 4:** Pearson correlation coefficient between MFVS and MFVs of different traits.

Trait	Correlation Coefficient
SFW	0.730 *
RFW	0.621 *
SDW	0.484 *
RDW	0.633 *
SL	0.588 *
RL	0.139
RWC	0.214
Pro	0.102
Sug	−0.074
Chl	0.433
Cart	−0.072
Na^+^	−0.083
K^+^	0.674 *
K^+^/Na^+^	0.278

* Significant at 5% as determined by the *t*-test.

**Table 5:** Multiple linear stepwise regression of total MFVS using MFVs of individual traits as predictors in hybrids and parents.

Independent Variable	Coefficient (B)	Standard Error	t	*p* Value
Intercept	0.170	0.036	4.728	***
SFW	0.348	0.051	6.840	***
Pro	0.165	0.043	3.828	**
R^2^	0.772			
Adjusted R^2^	0.740			
Cp	3.000			

**, *** significant at 1% and 0.1%, respectively, as determined by the F-test.

**Table 6:** General combining ability effects of parents under control conditions.

	L1	L2	T1	T2	T3	T4	T5
SFW	−73.69	73.69	143.89	160.03	80.12	110.41	174.40
RFW	−4.75	4.75	−16.43 **	−10.48 *	2.55	9.02 *	15.34 *
SDW	−0.11	0.11	0.75	2.28 *	−0.67	−1.18	0.32
RDW	−13.11	13.11	−223.68	308.49	−211.29	40.48	86.02
SL	0.33 *	−0.33 *	0.42	−0.12	0.30	−0.05	0.05
RL	0.18	−0.18	−0.33	−0.05	0.38	0.05	−0.05
RWC	3.72	−3.72	−225.74	339.26	−175.40	199.86	261.74
Pro	−14.32	14.32	−220.25	102.98	61.75	−229.76	285.28
Sug	54.09	−54.09	−162.65	−167.52	364.21	−165.62	131.59
Chl	7.14	7.14	117.45	−60.67	12.45	112.02	−151.25
Cart	0.15 **	−0.15 **	−23.28 **	3.37 **	2.33 **	−4.28 **	21.85 **
Na^+^	−0.08 **	0.08 **	−0.12 **	−0.05 **	−0.25 **	0.69 **	−0.27 **
K^+^	0.11 **	−0.11 **	−0.01	0.09 **	0.16 **	−0.25 **	0.02
K^+^/Na^+^	−0.16 *	0.16 *	−0.14	0.56 **	1.50 **	−2.37 **	1.57 **

*, ** significant at 5% and 1%, respectively, as determined by the *t*-test.

**Table 7:** General combining ability effects of parents under salt-stress conditions.

	L1	L2	T1	T2	T3	T4	T5
SFW	2.17	−2.17	8.66	−21.29 **	8.76	2.83	1.04
RFW	−0.47	0.47	3.25	−7.08 **	3.05	0.07	0.72
SDW	−0.07	0.07	0.04	0.04	0.17	−1.33	1.09
RDW	−0.15	0.15	0.30	0.28	−0.00	−0.40	−0.18
SL	0.16	−0.16	0.31	−0.40	−0.27	−0.19	0.55 *
RL	0.12	−0.12	0.34 **	−0.38 **	0.27 *	−0.14	−0.09
RWC	0.44	−0.44	−0.07	−0.96	−0.04	1.13 *	−0.06
Pro	−254.81 **	254.81 **	31.74 *	310.88 **	−184.55 **	−123.48 **	−34.59
Sug	0.36 **	−0.36 **	1.41 **	4.57 **	−5.69 **	−1.76 **	1.46 **
Chl	52.41 **	−52.41	117.47 **	−171.81 **	−81.36 **	−21.50 **	157.19 **
Cart	−2.34	2.34	1.68	−5.65	4.90	3.98	−4.90
Na^+^	−0.06 **	0.06 **	0.84 **	0.58 **	−0.84 **	−1.02 **	0.44 **
K^+^	−0.09 **	0.09 **	−0.06 *	0.04	−0.03	0.02	0.03
K^+^/Na^+^	−0.00	0.00	−0.08 **	−0.05 **	0.06 **	0.12 **	−0.04 **

*, ** significant at 5% and 1%, respectively, as determined by the *t*-test.

**Table 8:** Estimates of genetic components of the total variance for the studied traits. σ^2^_GCA_—general combining ability variance, σ^2^_SCA_—specific combining ability variance, σ^2^_A_—additive genetic variance, σ^2^_D_—dominance genetic variance.

	Control		Salinity	
	σ^2^_GCA_/σ^2^_SCA_	σ^2^_A_/σ^2^_D_	σ^2^_GCA_/σ^2^_GCA_	σ^2^_A_/σ^2^_D_
SFW	−0.69	0.90	0.90	0.45
RFW	0.76	−0.04	−0.04	−0.04
SDW	−0.01	−0.07	−0.07	−0.07
RDW	−0.03	−0.08	−0.08	−0.08
SL	0.04	0.62	0.62	0.45
RL	−0.77	−0.02	−0.02	−0.02
RWC	−0.09	−0.06	−0.06	−0.06
Pro	−0.01	−0.06	−0.06	−0.06
Sug	0.08	−0.03	−0.03	−0.03
Chl	0.05	−0.05	−0.05	−0.05
Cart	−0.02	−0.07	−0.07	−0.07
Na^+^	0.05	0.03	0.03	0.15
K^+^	−0.02	−0.04	−0.04	−0.02
K^+^/Na^+^	0.10	−0.01	−0.01	−0.01

**Table 9:** Name, source and code of oasis landraces, local varieties and their corresponding hybrids.

	Code	Name	Origin	Hybrid	Code
Testers ♂	T1	Oum Rokba El Baida	Touat	Florence-aurore × Oum Rokba El Baida	H1
	T2	Chater	Touat	Florence-aurore × Chater	H2
	T3	Oum Rokba Elhamra	Touat	Florence-aurore × Oum Rokba Elhamra	H3
	T4	Khellouf	Oued Righ	Florence-aurore × Khellouf	H4
	T5	Zeghlou	Touat	Florence-aurore × Zeghlou	H5
Lines ♀	L1	Florence-aurore	Tunisia	Ain abid × Oum Rokba El Baida	H6
	L2	Ain abid	Spain	Ain abid × Chater Ain abid × Oum Rokba Elhamra Ain abid × Khellouf Ain abid × Zeghlou	H7 H8 H9 H10

## Data Availability

Data are contained within the article and Supplementary File.

## Supplementary Materials

The following supporting information can be downloaded at: https://www.mdpi.com/article/10.3390/plants14060911/s1, Table S1: Mean squares of Line × Tester ANOVA of bread wheat seedlings under control conditions.; Table S2: Mean squares of Line × Tester ANOVA of bread wheat seedlings under salt stress conditions.

## Abbreviations

The following abbreviations are used in this manuscript

Chl	Chlorophyll content
RWC	Relative water content
Pro	Proline content
Sug	Total soluble sugars content
Cart	Catenoids content
Na^+^	Sodium ions content
K^+^	Potassium ions content
K^+^/Na^+^	Selectivity of potassium ions over sodium ions
SFW	Shoot fresh weight
RFW	Root fresh weight
SDW	Shoot dry weight
RDW	Root dry weight
SL	Shoot length
RL	Root length
MFVS	Membership function value of salt tolerance
H_MP_	Mid-parent heterosis
H_BP_	Better-parent heterosis
SCA	Specific combining ability
GCA	General combining ability
EC	Electrical conductivity
NaCl	Sodium chloride
