# Supplementary material for: Inheritance of Some Salt Tolerance-Related Traits in Bread Wheat (Triticum aestivum L.) at the Seedling Stage: A Study of Combining Ability
Source: Plants (Basel). 2025 Mar 14;14(6):911. doi: 10.3390/plants14060911 (PMC11944529; doi:10.3390/plants14060911)
Supplement: Supplementary file 1 [file plants-14-00911-s001.zip › plants-3420588-supplementary.pdf]

Supplementary table S1. Mean squares of Line ×Tester ANOVA of bread wheat seedlings under control conditions.

	replication	genotype	Parents	Parents vs crosses	crosses	Testers	Lines	L x T	Error
**Df**	2	16	6	1	9	4	1	4	32
**SFW**	1.16	2255.34**	3640.85**	15.20	1580.58*	2385.15	164.97	1130.18	657.42
**RFW**	29.91	490.45**	458.95**	248.53	538.33**	853.22*	976.41*	113.92	85.80
**SDW**	0.170	23.31**	43.98**	1.59	11.95	13.17	1.51	13.34	6.19
**RDW**	0.87	5.15**	4.88**	11.06**	4.67**	3.23	2.79	6.57**	0.63
**SL**	0.16	0.78**	0.71*	0.21	0.89**	1.36**	2.35**	0.062	0.22
**RL**	0.44	2.61**	4.99**	1.20	1.18*	1.64	0.93	0.79	0.49
**RWC**	0.14	1.40**	1.34**	2.28**	1.34**	1.68	0.018	1.33**	0.19
**Pro**	1.47	6313.93**	4569.31**	899.27**	8078.64**	13596.6	27.37	4573.43**	16.32
**Sug**	3.06**	2.57**	2.39**	2.15**	2.95**	2.54	5.78	2.66**	1.16
**Chl**	7.54**	1.62**	1.73**	1.03**	5.79**	3.46	3.66	8.65**	4.75
**Cart**	3.57**	3.06**	5.39	3.17**	1.49**	1.72	3.44	1.55**	1.34
**Na+**	0.007*	0.43**	0.024**	1.02**	0.64**	0.37	0.38	0.97**	0.001
**K+**	0.03**	0.18**	0.11**	0.30**	0.21**	0.33	0.13	0.11**	0.003
**K+/Na+**	1.36**	7.2**	3.07**	18.02**	9.67**	2.13	6.18	18.07**	0.10

*, ** significant at 5% and 1%, respectively, as determined by the F-test

Supplementary table S2. Mean squares of Line ×Tester ANOVA of bread wheat seedlings under salt stress conditions.

	replication	genotype	Parents	Parents vs crosses	crosses	Testers	Lines	L x T	Error
**Df**	2	16	6	1	9	4	1	4	32
**SFW**	147.35	848.05**	1376.47**	765.93*	504.90**	106.22	5.29	1028.48**	137.52
**RFW**	0.14	130.51**	63.57*	654.00**	116.98**	7.29	280.90	185.69**	23.05
**SDW**	2.89	3802.43**	20.68**	60621.68**	10.68**	4.52	0.07	19.49**	2.25
**RDW**	0.60	1.26**	1.74**	0.29	1.05*	0.53	0.630	1.68*	0.42
**SL**	2.53*	0.53	0.37	0.05	0.69	0.63	0.04	0.90	0.53
**RL**	0.17	0.43**	0.28*	0.002	0.58**	0.74	0.67	0.41**	0.08
**RWC**	0.13	12.62**	20.30**	0.22**	8.87**	4.44	0.08	15.51**	1.18
**Pro**	34.7×10^{3}*	24.2×10^{5}*	36×10^{5}**	20.9×10^{5}**	16.8×10^{5}**	94.9×10^{4}	27.5×10^{4}	27.7×10^{5}**	10.1×10^{2}
**Sug**	2.22**	118.13**	177.78**	16.00**	89.71**	107.89	98.42	69.35**	0.26
**Chl**	7.54**	1.20**	2.54**	2.03**	1.97**	2.63	3.42	9.49**	2.44
**Cart**	3.57**	6.48**	1.50**	3.98**	1.03**	5.85	2.72	1.68**	8.82
**Na^+**	0.02	3.46**	2.59**	11.02**	3.20**	4.44	6.96	1.02	0.05
**K^+**	0.039**	0.069**	0.07**	0.02*	0.07**	0.12	0.03	0.02**	0.002
**K^+/Na^+**	0.001*	0.02**	0.009**	0.04**	0.036**	0.04	0.05	0.02**	0.00

*, ** significant at 5% and 1%, respectively, as determined by the F-test
